# Targeting PRMT5 enhances the radiosensitivity of tumor cells grown in vitro and in vivo

**DOI:** 10.1038/s41598-024-68405-8

**Published:** 2024-07-27

**Authors:** Charlotte Degorre, Steven Lohard, Christina N. Bobrek, Komal N. Rawal, Skyler Kuhn, Philip J. Tofilon

**Affiliations:** 1 Radiation Oncology Branch, National Cancer Institute, 10 Center Drive-MSC 1002, Building 10, B3B69B, Bethesda, MD 20892 USA; 2 Integrated Data Sciences Section, Research Technologies Branch, National Institute of Allergy and Infectious Diseases, National Institutes of Health, Bethesda, MD USA

**Keywords:** PRMT5, Radiosensitization, DNA damage response, Cancer, Cell biology, Molecular medicine

## Abstract

PRMT5 is a widely expressed arginine methyltransferase that regulates processes involved in tumor cell proliferation and survival. In the study described here, we investigated whether PRMT5 provides a target for tumor radiosensitization. Knockdown of PRMT5 using siRNA enhanced the radiosensitivity of a panel of cell lines corresponding to tumor types typically treated with radiotherapy. To extend these studies to an experimental therapeutic setting, the PRMT5 inhibitor LLY-283 was used. Exposure of the tumor cell lines to LLY-283 decreased PRMT5 activity and enhanced their radiosensitivity. This increase in radiosensitivity was accompanied by an inhibition of DNA double-strand break repair as determined by γH2AX foci and neutral comet analyses. For a normal fibroblast cell line, although LLY-283 reduced PRMT5 activity, it had no effect on their radiosensitivity. Transcriptome analysis of U251 cells showed that LLY-283 treatment reduced the expression of genes and altered the mRNA splicing pattern of genes involved in the DNA damage response. Subcutaneous xenografts were then used to evaluate the in vivo response to LLY-283 and radiation. Treatment of mice with LLY-283 decreased tumor PRMT5 activity and significantly enhanced the radiation-induced growth delay. These results suggest that PRMT5 is a tumor selective target for radiosensitization.

## Introduction

Approximately 50% of patients with solid tumors receive radiotherapy during their treatment^1^. Towards increasing the efficacy of radiation therapy, one approach has been the development of targeted radiosensitizers, which requires identifying the molecular determinants of tumor cell radioresponse. Along these lines, PRMT5 is a widely expressed type II arginine methyltransferase that symmetrically dimethylates histone and non-histone proteins. PRMT5 mediated arginine methylation of histone tails results in the activation or repression of target gene transcription depending on the chromatin environment^2^. In addition, PRMT5 methylates proteins associated with the spliceosome, facilitating mRNA splicing^3–5^. While PRMT5 has additional substrates involved in such processes as ribosome biogenesis and cell signaling^2,6^, its primary impact on cell biology appears to be as an epigenetic regulation of gene expression largely mediated via effects on transcription and mRNA splicing^2^.

PRMT5 is overexpressed in a variety of human cancers including leukemia and lymphoma^7^, gastric cancer^8^, breast cancer^9^, colorectal cancer^10^ and glioblastoma^11,12^, which is correlated with a poor prognosis^13,14^. Preclinical studies have shown that inhibition of PRMT5 activity suppresses tumor cell proliferation in vitro and in animal models slows tumor growth and prolongs survival, suggesting that PRMT5 provides a target for cancer therapy^15,16^. PRMT5 has also been implicated as a regulator of the DNA damage response (DDR). In a panel of human tumor cell lines PRMT5 expression positively correlated with the expression of DDR related genes^17^. In the same study, the PRMT5 inhibitor C220 reduced the transcription and altered the splicing pattern of selected DDR genes and enhanced tumor cell sensitivity to the DNA damaging chemotherapeutic agents cisplatin, olaparib and 5-FU. Braun et al. showed that the mRNA splicing changes induced by PRMT5 inhibition occurred in genes participating in DNA repair and cell cycle regulation^18^. Loss of PRMT5 activity through depletion or inhibition in leukemic cells resulted in the aberrant splicing of the DNA repair gene TIP60/KAT5 leading to impaired homologous recombination repair and enhanced sensitivity to the olaparib^19^. Thus, operating through epigenetic control of gene expression, PRMT5 appears to enhance the ability of cells to survive DNA damage. Of note, PRMT5 can also directly influence the activity of specific components of DNA repair processes. Specifically, arginine methylation of tyrosyl-DNA phosphodiesterase by PRMT5 enhances the removal topoisomerase I cleavage complexes and the repair of DNA single strand breaks to protect against camptothecin induced cell death^20,21^.

The putative role of PRMT5 in the DDR then suggests that it may function as a determinant of cellular radiosensitivity. To investigate the potential of PRMT5 to serve as a target for radiosensitization, we used a panel of cell lines corresponding to tumor types typically treated with radiotherapy. The data shown here indicate that loss of PRMT5 activity selectively enhances tumor cell radiosensitivity through an inhibition of DNA double strand break (DSB) repair. Consistent with these findings and as in previous studies^17,19^, PRMT5 inhibition reduced expression and altered the mRNA splicing pattern of genes involved in the DDR. Finally, PRMT5 inhibition in vivo enhanced the radiation-induced growth delay of two types of subcutaneous tumor xenografts.

## Material and methods

### Cell lines and treatment

The U251 glioblastoma (GBM) cell line was obtained from the division of cancer treatment and diagnosis tumor repository (DCTD, National Cancer Institute). PSN1 (Pancreatic adenocarcinoma), MDA-MB-231 (Breast adenocarcinoma) and MRC9 (normal lung fibroblasts) were purchased from American type culture collection (ATCC). DMEM supplemented with 10% FBS (Invitrogen) was used to culture the U251 and MDA-MB-231 cell lines. PSN1 and MRC9 were respectively cultured in RPMI-1640 supplemented with 10% FBS, and MEM supplemented with 10% FBS, sodium pyruvate, L-glutamine, and nonessential amino acids (all from Invitrogen). All cell lines were maintained at 37 °C in an atmosphere of 5%CO_2_/95% air. Each cell line was cultured for a maximum of 2 months and tested negative for mycoplasma contamination by mycoplasma alert assay (Lonza Inc). LLY-283 was obtained from Sellechchem and for in vitro treatment, dissolved in dimethyl sulfoxide (DMSO). Cells were irradiated using a 320 kV X-ray source (Precision X-ray, Inc.) with a 2.0 mm aluminum filtration (300 kV peak, 10 mA) at a dose rate of 2 Gy/minute.

### siRNA transfection

At 60–80% confluence, tumor cells were transfected with a pool of 4 targeted siRNA against PRMT5 or 4 non-targeting siRNA (Dharmacon) using the Lipofectamin RNAiMAX (Invitrogen) according to the manufacturer’s protocol. Experiments were all performed 48 h post-transfection.

### Immunoblot analysis

Cells were lysed in 50 mmol/L Tris–HCl (pH 7.5), 150 mmol/L NaCl, 2 mmol/L EDTA, 2 mmol/L EGTA, 0.2% Triton X-100, 0.3% Tween20 supplemented with 1 × phosphatase inhibitor cocktails II and III (Sigma-Aldrich), and 1 × HALT protease inhibitor cocktail (Thermo Scientific). Cell lysates were incubated for 2 h on ice and centrifuged for 30 min at 1200 rpm at 4° C. Total protein was quantified by BCA assay (Thermo Scientific) and 40 µg of protein were separated by SDS-PAGE and transferred on a nitrocellulose membrane (Biorad). Proteins were blocked using 5% non-fat dry milk (Biorad) for 1 h and membranes were hybridized overnight at 4° C to primary antibodies corresponding to PRMT5 (CS7998, Cell signaling), sDMA (CS13222, Cell signaling) or Actin (MAB1501, Millipore) and then 1 h with an HRP-coupled secondary antibody (Cell signaling). Proteins of interest were visualized with Pierce ECL Western Blotting substrate (Thermo Scientific) and the ChemiDoc MP imaging system (Biorad).

### sDMA ELISA

Levels of sDMA were determined using the ELISA analysis as described by Jensen-Pergakes et al.^22^. For in vitro experiments, cells were lysed in 50 mmol/L Tris-HCl (pH 7.5), 150 mmol/L NaCl, 2 mmol/L EDTA, 2 mmol/L EGTA, 0.2% Triton X-100, 0.3% Tween20 supplemented with 1 × phosphatase inhibitor cocktails II anD III (Sigma-Aldrich), and 1 × HALT protease inhibitor cocktail (Thermo Scientific). For in vivo experiments, subcutaneous tumors were dissected from the mouse leg and snap frozen in liquid nitrogen. Samples were homogenized (Polytron Brinkman) in the lysis buffer described above. Cells and tissue lysis were incubated for 2 h on ice and centrifuged for 30 min at 1200 rpm at 4° C. Total protein was quantified by BCA assay (Thermo Scientific) and 200 ng of protein were coated overnight at 4° C in a white flat bottom 96 well plate. Proteins were blocked using 5% Bovine Serum Albumin for 2 h, incubated with either sDMA (CS13222, Cell signaling) or SmD3 (AP12451a, Abcepta) antibodies overnight at 4° C and then 1 h at room temperature with an HRP-coupled secondary antibody (Cell signaling). Chemiluminescence was measured after incubation in the Supersignal ELISA Pipco Luminol (1856155, Thermo scientific) for 1 min, using the Synergy HL microplate reader (BioTek).

### Clonogenic survival

Radiosensitivity was determined using a colony forming efficiency assay. 48 h after transfection with PRMT5 siRNA, cells were plated at clonal density in 6 well plates and irradiated the next day. For the pharmacological inhibition, cells were seeded at clonal density, treated with LLY-283 or vehicle (DMSO) for 1 h, irradiated, and 24 h later LLY-283 was rinsed off and cultures were fed with fresh drug-free media. Ten to 21 days after radiation, colonies were stained with 0.5% crystal violet solution in methanol and the number of colonies containing at least 50 cells determined allowing to calculate the survival fraction. Radiation survival curves were generated after normalizing to the cytotoxicity induced by PRMT5 knockdown or LLY-283.

### RNA isolation and sequencing

For transcriptome analysis, U251 cells were treated with LLY-283 (100 nM) or vehicle for 24 h. RNA was extracted with the RNeasy mini kit (Qiagen) according to the manufacturer protocol. RNA-seq was performed on the purified RNA by the Center for Cancer Research Sequencing Facility in Frederick, Maryland. Briefly, 200 ng of RNA was used as input for mRNA capture with oligo-dT coated magnetic beads. The mRNA was fragmented, followed by random-primed cDNA synthesis. The resulting double-stranded cDNA was used as the input to a standard Illumina library prep with end-repair, adapter ligation and PCR amplification to generate a sequencing ready library. The final cDNA libraries were quantitated by qPCR before cluster generation and sequenced on an Illumina NextSeq.

### RNA-seq analysis

The raw base calls were demultiplexed and converted into FastQ format using bcl2fastq. The raw FastQ files were processed using version 1.7.1 of the OpenOmics/RNA-seek pipeline (https://github.com/OpenOmics/RNA-seek). This pipeline orchestrates a comprehensive set of data-processing and quality-control steps. Briefly, the quality of each sample was assessed using FastQC v0.11.9 (https://www.bioinformatics.babraham.ac.uk/projects/fastqc/.), Preseq v2.0.3^23^, Picard tools v2.17.11 (https://broadinstitute.github.io/picard/), FastQ Screen v0.9.3^24^, Kraken2 v2.0.8^25^, QualiMap^26^, and RSeQC v2.6.4^27^. Adapter sequences were trimmed using Cutadapt v1.18^28^. The trimmed reads were mapped against a GENCODE^29^ human primary assembly, GRCh38.p13, index using the splice-aware aligner STAR version 2.7.6a^30^. Gene and transcript expression levels were estimated with GENCODE’s comprehensive human gene annotation (release 41) via RSEM v1.3.3^31^. Low count genes were filtered from the raw count matrix prior to performing any downstream analysis. Differential gene expression analysis was performed using the R package limma^32^. rMATS v4.1.1^33^ was used to identify alternative splicing events. The data discussed in this publication have been deposited in NCBI’s gene expression omnibus^34^ and are accessible through GEO Series accession number GSE264609 (https://www.ncbi.nlm.nih.gov/geo/query/acc.cgi?acc=GSE264609). Genes with an adjusted p-value < 0.05 were submitted for ingenuity pathway analysis (IPA) (QIAGEN). Genes were also ranked by (sign of the fold change) *−log10(p-value) and submitted to GSEA^35^ for pre-ranked analysis with 1000 permutations against the GO database.

### γH2AX foci formation

γH2AX foci were analyzed as previously described^36^ using antibodies against phosphorylated H2AX (Millipore Sigma #05–636) followed by anti-mouse IgG Alexa Fluor 488 and DAPI (Invitrogen). Slides were imaged on a Zeiss Axio Imager 2 with a 40 × oil immersion lens. γH2AX foci were counted in 50 cells per treatment group.

### Neutral comet assay

Cells were treated with either LLY-283 (100 nM) or vehicle (DMSO) for 1 h, irradiated (10 Gy) and returned to the incubator. At specified times, single-cell suspensions were generated, washed with PBS, and mixed with low melting agarose (1:10). The resulting mixture was transferred to the provided slides of the commercial kit (Trevigen) and allowed to solidify for 10 min at 4 °C. Cells were then lysed for 1 h on ice, subjected to electrophoresis at 1 V/cm for 20 min at room temperature and fixed with 70% EtOH. After staining the DNA with SYBR Green, the slides were imaged within an hour using a Zeiss Axio Imager 2 with a 5 × objective. The digital fluorescent images were analyzed with TriTek CometScore as described in^37^. Data are expressed as percent DNA damage remaining in which the tail moment immediately after irradiation corresponds to 100% damage. At least 50 cells per condition were measured.

### Tumor growth delay study

A single cell suspension of 2 × 10^6^ U251 or 5 × 10^6^ PSN1 cells was implanted subcutaneously in the right hind leg of seven- to eight-week-old athymic female nude mice (NCr nu/nu; NCI Animal Production Program). On day 13 or 5 post-implantation for U251 or PSN1 respectively, mice were randomized according to the tumor volume into 4 groups, with each group having an average volume of around 200 mm^3^. LLY-283 was dissolved in 1% hydroxyethyl cellulose (HEC), 0.25% Tween-80, 0.05% antifoam in ultrapure water. Each treatment group contained 6 and 8 mice for U251 and PSN1, respectively. Radiation (3 Gy) was delivered for 3 consecutive days to animals restrained in a custom designed lead jig using an X-Rad 320 X-irradiator (Precision X-Rays, Inc.) with a 2.0 mm aluminum filtration (320 kV, 12.5 mA, 3.39 Gy/min dose rate). LLY-283 (100 mg/kg) or vehicle was administrated by oral gavage 24 h before each irradiation. Tumor measurements were taken twice a week with a digital caliper, and tumor volumes were calculated using the formula (length x width^2^)/2. GraphPad Prism10 was used to determine the percent of mice with tumors less than 1000mm^3^ as a function of time after treatment with log-rank tests for significance. All animal studies were conducted in accordance with the principles and procedures outlined in the NIH Guide for Care and Use of Animals and approved by the Institutional Animal Care and Use Committee (IACUC).

### Statistics

For in vitro experiments, statistical significance was determined using a two-tailed Student t-test. For in vivo studies, Kaplan–Meier curves were generated, and log-rank values calculated in GraphPad Prism 10 (GraphPad Software).

## Results

**Figure 1:**
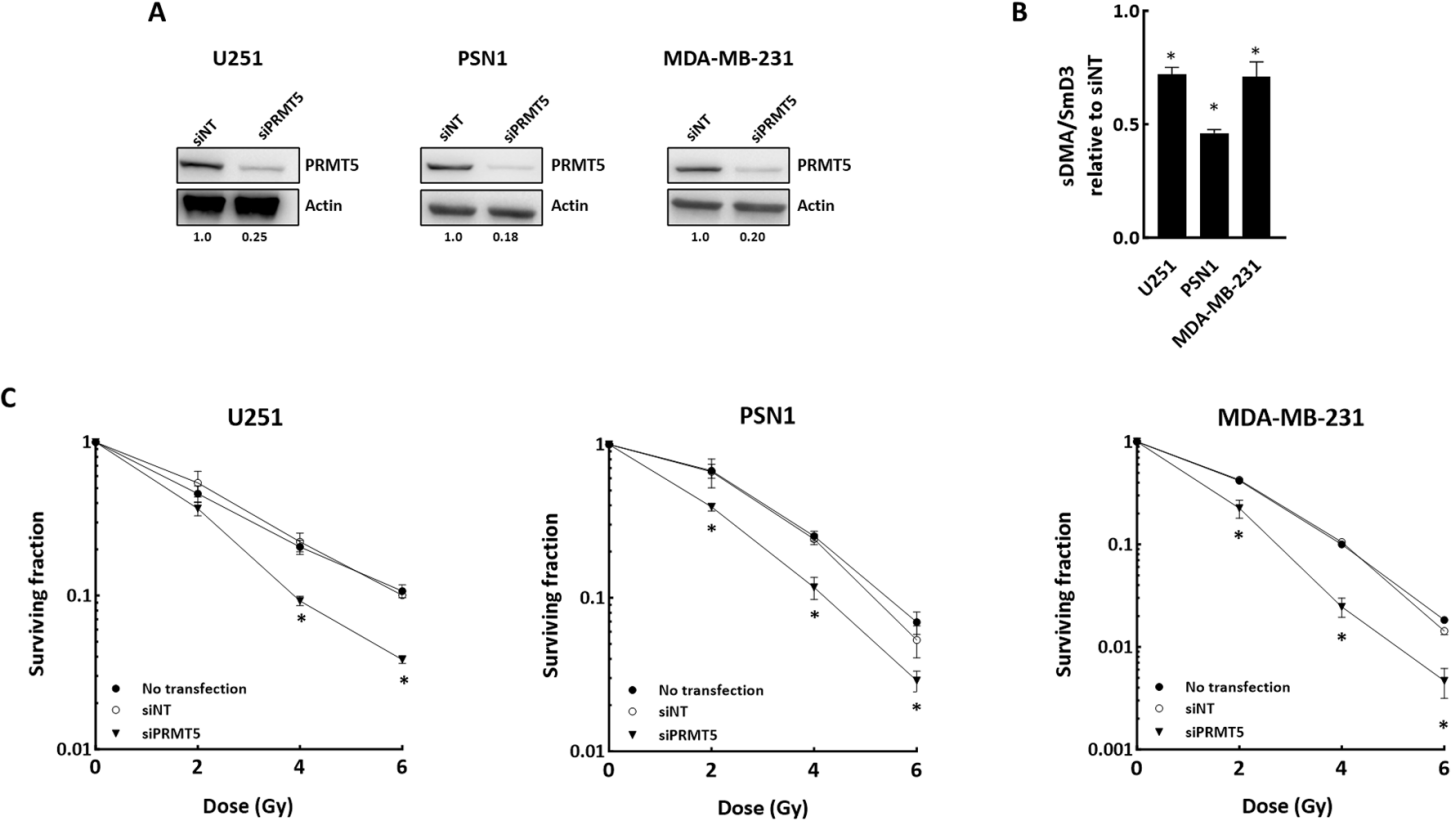
Effects of PRMT5 knock-down on the radiosensitivity of tumor cell lines. (**A**) Tumor cell lines were transfected with non-targeting siRNA (siNT) or siRNA against PRMT5 (siPRMT5). 48 h post-transfection, cell lysates were immunoblotted for PRMT5 and actin as a loading control. Normalized ratios of PRMT5 to actin are shown below blots. Uncropped gels are shown in supplemental Figure S1. (**B**) 48 h post transfection, cell lysates were subjected to ELISA for sDMA and SmD3 with data expressed as sDMA normalized by SmD3 and relative to the vehicle. Values represent the mean ± SEM for three independent experiments. (**C**) Clonogenic survival analysis on U251, PSN1 and MDA-MB-231. Forty-eight hours post-transfection, cells were plated at clonal density, irradiated 16 h later and colonies stained 10–24 days post-radiation. DEFs were calculated at a surviving fraction of 0.1. Values represent the mean ± SEM for 3 independent experiments. *p < 0.05 by Student’s t-test.

To determine whether PRMT5 influences the radiosensitivity of solid tumor cells, U251 (glioblastoma), PSN1 (pancreatic carcinoma) and MDA-MB-231 (breast adenocarcinoma) cell lines were transfected with either non-targeted siRNA pool (siNT) or siRNA pool specific to PRMT5 (siPRMT5); 48 h after transfection cells were subjected to analyses. As shown in Fig. 1A, siPRMT5 treatment of U251, PSN1 and MDA-MB-231 decreased the level of PRMT5. This decrease in PRMT5 protein corresponded to a decrease in the overall activity of PRMT5, as indicated by the reduction in symmetric dimethyl arginine (sDMA)^22^ levels detected by ELISA (Fig. 1B). The reduction in PRMT5 activity as detected by ELISA was corroborated by immunoblot analysis of sDMA levels after siPRMT5 treatment (Supplementary Figure S2). To determine the effects of PRMT5 knockdown on radiosensitivity, cells were transfected as described above, trypsinized and irradiated 16 h after seeding at clonal density. Transfection with siRNA to PRMT5 resulted in a significant enhancement of radiosensitivity in all three tumor cell lines U251, PSN1 and MDA-MB-231, with DEFs (Dose Enhancement Factor at a surviving fraction of 0.1) of 1.54, 1.41 and 1.53, respectively (Fig. 1C). Surviving fractions after knockdown of PRMT5 alone for U251, PSN1 and MDA-MB-231 were 0.96 ± 0.063, 0.66 ± 0.27 and 0.17 ± 0.04, respectively compared to the siNT. These results indicate that PRMT5 contributes to tumor cell survival after irradiation.

**Figure 2:**
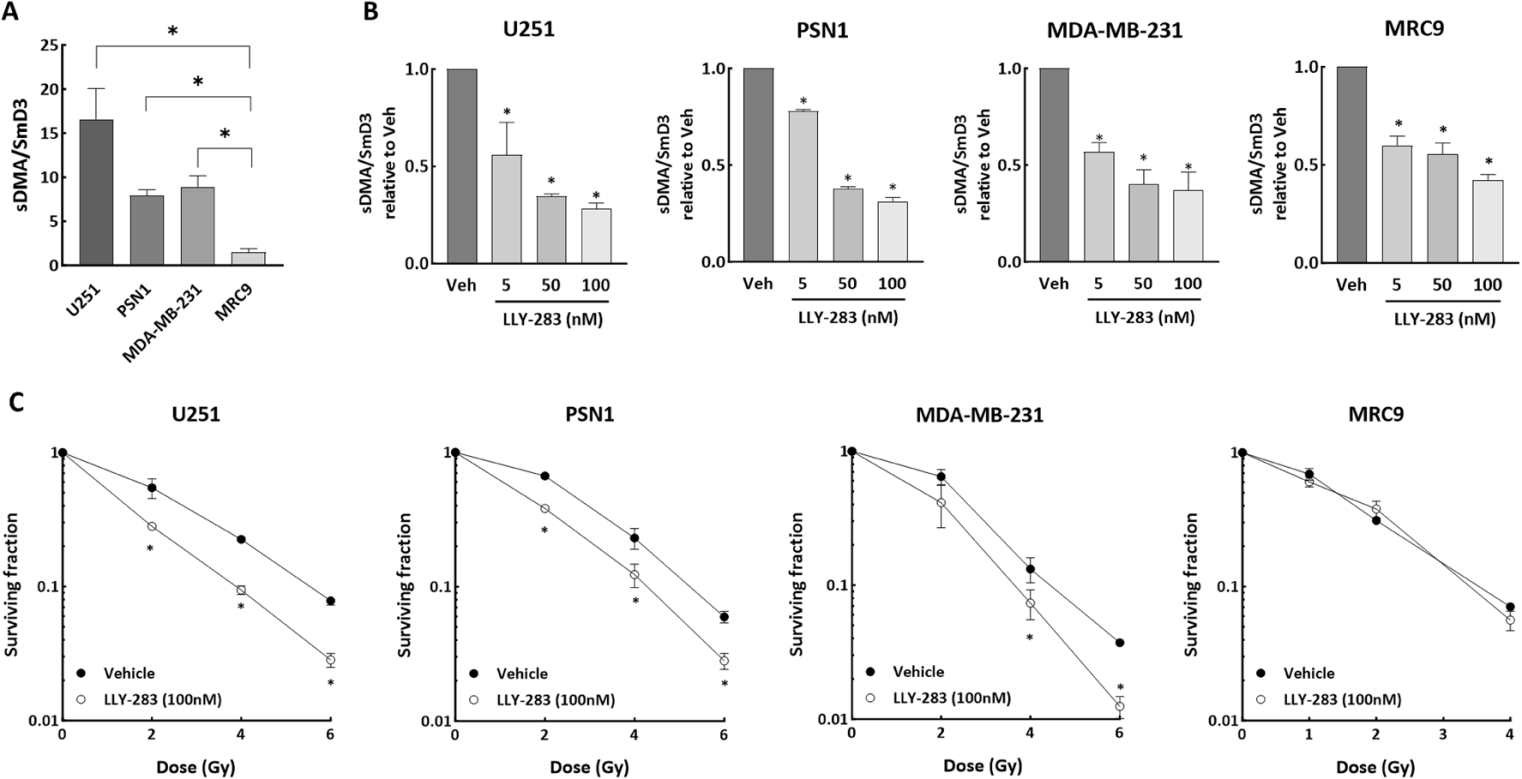
Effects of LLY-283 on PRMT5 activity and radiosensitivity. (**A**) sDMA levels in untreated cell lines. Data are expressed as the level of sDMA normalized by SmD3. Values represent the mean ± SEM for three independent experiments. (**B**) sDMA levels at 24 h after LLY-283 treatment. Cells were treated with increasing dose of LLY-283 (5-100 nM) for 24 h and subjected to ELISA. Data are expressed as the level of sDMA normalized by SmD3 and relative to vehicle. Values represent the mean ± SEM for three independent experiments. (**C**) U251, PSN1, MDA-MB-231 and MRC9 cell lines were plated at clonogenic density and treated with 100 nM of LLY-283 1 h before irradiation. 24 h post-irradiation, drug containing media was removed, replaced with drug-free media and colonies determined after 10–24 days post-irradiation. Values represent the mean ± SEM for three to four independent experiments. *p < 0.05 by Student’s t-test.

To extend this study to an experimental therapeutic setting, the small molecule LLY-283 was used to inhibit PRMT5 activity^15,38^. For this study, in addition to the tumor cell lines, the normal fibroblast cell line MRC9 was included. Comparison of sDMA levels in untreated cells showed that PRMT5 activity in the tumor cell lines was significantly higher than in the normal fibroblast cell line MRC9 (Fig. 2A). Treatment with LLY-283 (24 h) resulted in a similar dose dependent reduction of sDMA levels in the four cell lines indicating an inhibition of PRMT5 activity (Fig. 2B). To determine the effects of LLY-283 on radiosensitivity, cells were plated at clonal density and treated with LLY-283 (100 nM) 1 h prior to irradiation; 24 h later LLY-283 was removed from cultures by rinsing followed by feeding with fresh drug-free media. Treatment of the tumor cell lines with LLY-283 increased the level of radiation-induced cell killing, resulting in DEFs of 1.43, 1.22, and 1.23 for U251, PSN1, and MDA-MB-231, respectively (Fig. 2C). In contrast, LLY-283, although inhibiting PRMT5 activity, had no effect on the radiosensitivity of MRC9 normal fibroblasts. Treatment with LLY-283 (100 nM) alone did not induce cytotoxicity. These findings suggest that LLY-283 enhances tumor cell radiosensitivity but has no effect on the radiosensitivity of normal cells. Surviving fractions after treatment with LLY-283 alone for U251, PSN1, MDA-MB-231 and MRC9 were 0.95 ± 0.01, 1.22 ± 0.13, 1.29 ± 0.18, 0.78 ± 0.06, respectively.

**Figure 3:**
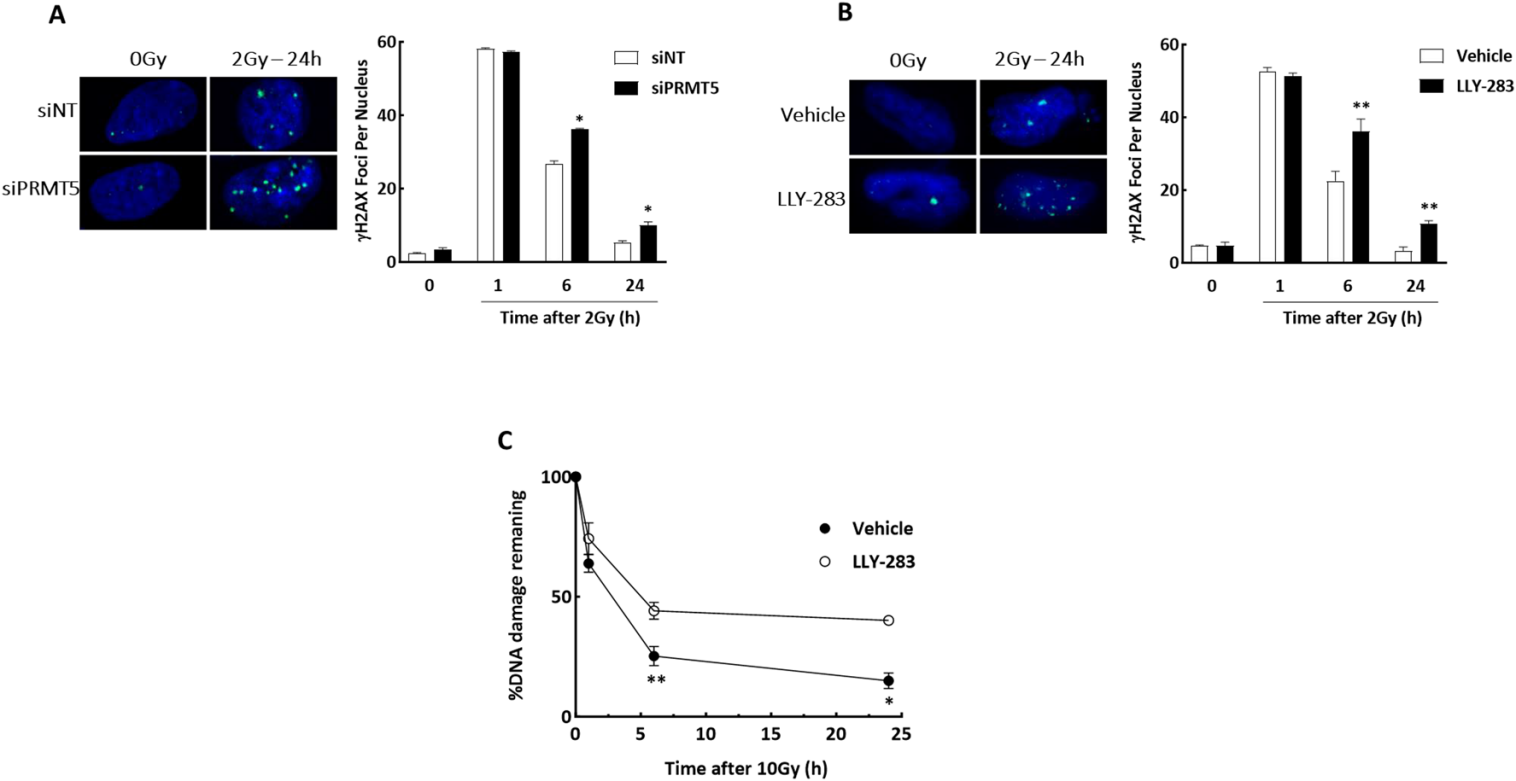
PRMT5 inhibition reduces DSB repair after radiation in U251 cell line. (**A**) siNT and siPRMT5 U251 cells were seeded 48 h post-transfection, irradiated (2 Gy) the next day, and collected at the indicated time points for foci analysis. γH2AX foci were counted in 50 cells per treatment group. Values represent the mean ± SEM for 3 independent experiments. (**B**) U251 cells were treated with 100 nM of LLY-283 for 1 h, irradiated (2 Gy), and collected at the indicated time points for foci analysis. γH2AX foci were counted in 50 cells per treatment group. Values represent the mean ± SEM for 3 independent experiments. (**C**) Neutral comet assay performed on U251 cells. U251 cells were exposed to LLY-283 1 h prior irradiation (10 Gy) and cells collected at the corresponding time points. Data are expressed as the percentage of DNA damage remaining as a function of time after 10 Gy in cells treated with LLY-283 or vehicle. Values represent the mean ± SEM for 3 independent experiments. *p < 0.05 by Student’s t-test.

As an initial investigation of the mechanism through which PRMT5 inhibition enhances radiosensitivity, DSB repair was evaluated according to the induction and dispersal of γH2AX foci. As shown in Fig. 3A, PRMT5 knockdown using siRNA had no effect on the radiation-induced γH2AX foci detected at 1 h, suggesting that loss of PRMT5 does not impact the initial level of DSBs. However, at 6 and 24 h after irradiation (2 Gy), the number of γH2AX foci was significantly higher in the siPRMT5 treated U251 compared to siNT. Treatment of U251 with LLY-283 (100 nM) 1 h prior to irradiation had a similar effect on radiation-induced γH2AX foci (Fig. 3B). No difference in γH2AX levels was detected at 1 h, whereas at 6 and 24 h after irradiation there was a significant increase in γH2AX foci detected in the LLY-283 treated cells. The persistence of radiation-induced γH2AX foci after PMRT5 knockdown and after exposure to LLY-283 suggests a decrease in DSB repair capacity. As an alternative measure of DSB repair, the neutral comet assay was performed on U251 cells treated with LLY-283 (100 nM) 1 h prior radiation (10 Gy) (Fig. 3C). The percentage of radiation-induced DSBs remaining at 6 and 24 h post-radiation was significantly greater in LLY-283 treated cells as compared to the vehicle. Thus, data generated from assays that measure different manifestations of DSBs suggest that a reduction in PRMT5 inhibits the repair of radiation induced DSBs.

**Figure 4:**
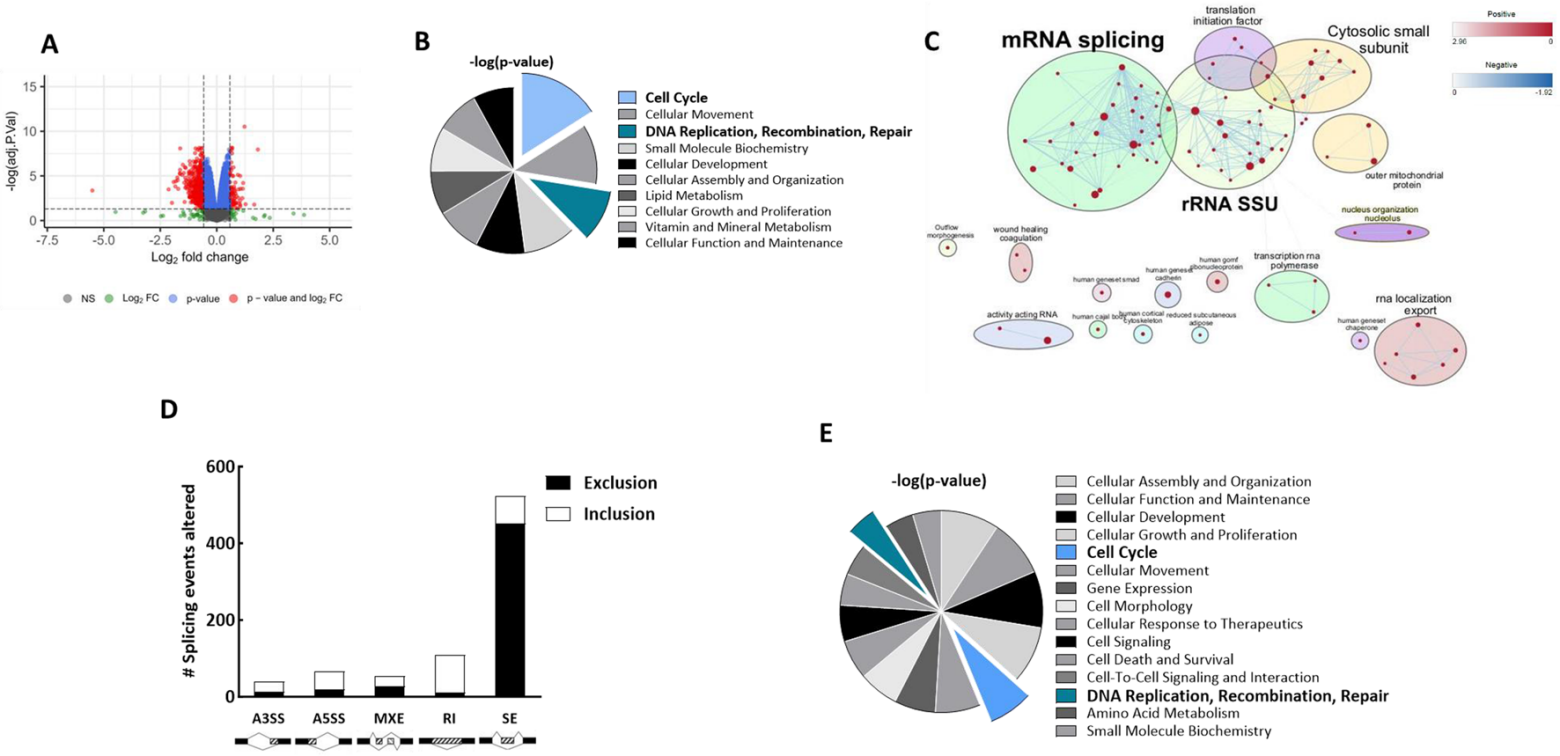
Effects of LLY-283 on gene expression and mRNA splicing. U251 cells were treated with LLY-283 (100 nM) for 24 h and analyzed by RNAseq (n = 4 replicates). (**A**) Volcano plot of genes affected by LLY-283. Genes significantly down and up regulated in LLY-283 vs vehicle are shown in red. (**B**) Pie chart of IPA defined top 10 significant “molecular and cellular functions” in LLY-283 differentially expressed genes. (**C**) Gene set enrichment analysis (GSEA) from the ranked gene list using the ontology gene set collection c5. An enrichment map was generated using Cytoscape (version 3.10.0). Within this network, significantly enriched gene ontology (GO) terms, identified by their significant normalized enrichment score (NES) (FDR < 0.05), are depicted as red (upregulated) or blue (downregulated) nodes connected by blue lines. Node size is correlated to the number of genes in the gene set and the blue lines represent shared genes between different gene sets. To enhance interpretation, the nodes have been circled and assigned biological function using the AutoAnnotate tools in Cytoscape. Additionally, individual node labels were removed for clarity using the publication-ready button in the EnrichmentMap section of Cytoscape. (**D**) Effects of LLY-283 on mRNA splicing in U251 cells. The number of splicing events (FDR < 0.05) altered by LLY-283 treatment is shown for each splice category. (**E**) Pie chart of IPA defined top 15 significant “molecular and cellular functions” for differentially expressed skipped exons (SE) genes.

To further evaluate potential mechanisms mediating LLY-283-induced radiosensitization, transcriptome analysis was performed on U251 cells. Specifically, cultures were treated with vehicle or LLY-283 (100 nM) for 24 h and subjected to RNAseq. As shown by the volcano plot in Fig. 4A, the expression of 83 genes was increased and 511 genes was decreased by LLY-283 treatment. The functional significance of these genes was evaluated using IPA (Fig. 4B); the top 10 significant “molecular and cellular functions” included *Cell cycle* and *DNA Replication, Recombination and Repair*, processes associated with the regulation of cellular radiosensitivity. To further evaluate the LLY-283-induced changes in gene expression, GSEA was performed using the ontology gene set collection c5 (Fig. 4C). An enrichment map was then created with Cytoscape using the significant Normalized Enrichment Score (NES) of each gene set. The map is organized as a network of gene sets clustered in corresponding biological functions. A significant enrichment in genes associated with the biological function “*mRNA splicing”* is observed in LLY-283 treated cells. This category encompasses 33 significantly upregulated gene sets (red node) with the highest NES correspond to functions such as *spliceosomal complex*, *RNA splicing* and *mRNA processing*, consistent with the previously reported effects of PRMT5 on the spliceosome^15,16^.

The effect of LLY-283 on mRNA splicing events in U251 cells was then evaluated using rMATS. Comparison of LLY-283 and vehicle revealed a total of 798 differentially spliced events (FDR < 0.05) occurring in 452 genes. The events included alternative splicing at the 3’ or 5’ site (A3/5SS), mutually exclusive exons (MXE), retained introns (RI) and skipped exons (SE), with the latter exhibiting the highest number of differential alternative splicing events (524 events from 321 genes) (Fig. 4D). Further analysis of the SE events revealed an 86% increase in the number of exon exclusion events in LLY-283 treated cells, suggesting that PRMT5 is required for exon definition and its inhibition severely alters the skipped exon process as previously reported^15,19,39^. Functional analysis using IPA performed on genes with LLY-283 mediated SE events showed that the top “molecular and cellular functions” included *Cell cycle* and *DNA Replication, Recombination and Repair* (Fig. 4E). These results suggest that LLY-283 modulates the expression as well as the splicing of genes participating in the DDR.

**Figure 5:**
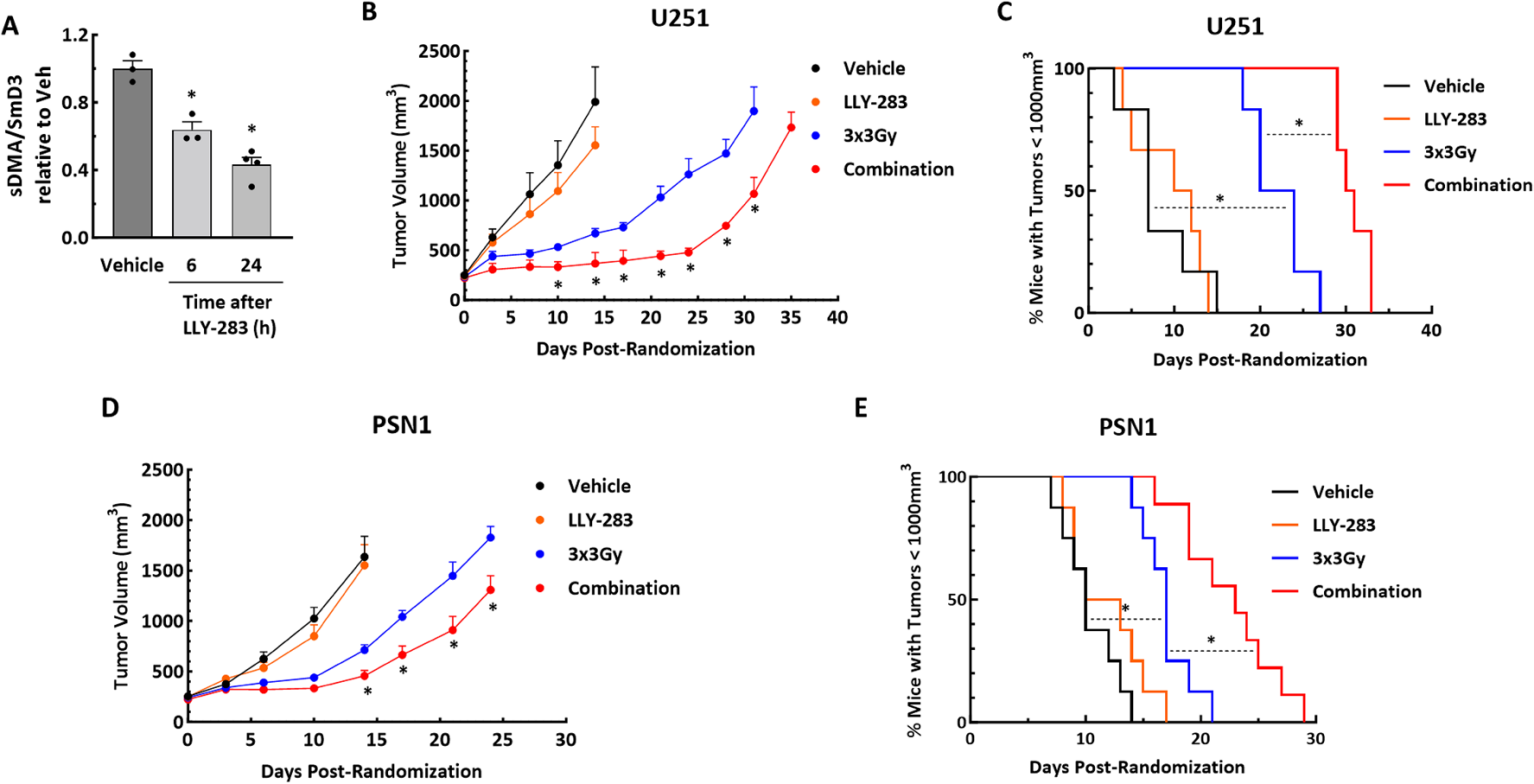
Effects of LLY-283 on PMRT5 activity and tumor radiosensitivity. (**A**) Mice bearing U251 subcutaneous xenografts were treated with vehicle or LLY-283 (100 mg/kg) by oral gavage. Tumors were collected at the indicated time points and cell lysates subjected to ELISA using sDMA and SmD3. The effects of LLY-283 are expressed as the level of sDMA normalized by SmD3. Values represent the mean ± SEM for three independent experiments. *p < 0.05 by Student’s t-test. (**B**) Mice bearing U251 leg tumors were treated with 3 Gy for 3 consecutive days and received LLY-283 (100 mg/kg) 24 h before each dose of radiation. Graph represents the average growth curves for each treatment group. Values represent the mean ± SEM for six mice. * p < 0.05 by Student’s t-test. (**C**) Kaplan–Meier graph represents the percent of mice with U251 tumors below 1000 mm^3^ at each measurement after treatment initiation. * p < 0.05 by log-rank (Mantel-Cox) test. (**D**) Mice bearing PSN1 leg tumors were treated with 3 Gy for 3 consecutive days and received LLY-283 (100 mg/kg) 24 h before each dose of radiation. Graph represents the average growth curves for each treatment group. Values represent the mean ± SEM for eight mice. *p < 0.05 by Student’s t-test. (**E**) Kaplan–Meier graph represents the percent of mice with PSN1 tumors below 1000 mm^3^ at each measurement after treatment initiation. *p < 0.05 by log-rank (Mantel-Cox) test.

To determine whether the LLY-283-induced tumor cell radiosensitization observed in vitro extends to an in vivo model, U251 cells were grown as subcutaneous xenografts. Initially, as an indicator of LLY-283 effectiveness in vivo, PRMT5 activity was assessed according to sDMA levels. Mice bearing U251 tumors were treated with a single dose of LLY-283 (100 mg/kg) by oral gavage and tumors were collected 6 and 24 h post treatment (Fig. 5A). A significant decrease of sDMA level was detected at 6 h with a further decrease at 24 h after LLY-283 treatment. Given these results, a treatment protocol was designed to investigate the antitumor effectiveness of the LLY-283/radiation combination. Mice bearing U251 xenografts were randomized into four treatment groups: Vehicle, LLY-283, radiation and LLY-283/radiation combination. A single dose of vehicle or LLY-283 (100 mg/kg) was administered via oral gavage 24 h before each radiation dose (3 Gy), which was delivered for three consecutive days. Growth curves showing the average tumor volumes at each time point are shown in (Fig. 5B) and the growth curves for the individual tumors are shown in Supplemental Figure S3. The time for the tumors to reach a volume of 1000 mm^3^ after the start of the treatment was evaluated for each individual mouse in all groups and used to calculate the absolute growth delay, which is defined as the time for treated tumors to reach 1000 mm^3^ minus the time for vehicle treated tumors to reach 1000 mm^3^. The absolute growth delay for LLY-283 alone was 1.3 ± 1.7 days (mean ± SEM), and radiation alone was 13.8 ± 1.4 days. The absolute growth delay for mice treated with the combination of LLY-283 and radiation was 22.5 ± 0.74, which is greater than the sum of the growth delays of the individual treatments. The DEF was calculated by subtracting the growth delay induced by LLY-283 alone (1.3) from the one observed in mice treated with the combination treatment (22.5); divided by the absolute growth delay of radiation alone (13.8) resulting in a DEF of 1.53. The tumor growth data were also evaluated according to the percent tumors less than 1000mm^3^ as determined on each day of measurement (Fig. 5C). Log-rank analysis showed that LLY-283 alone did not significantly modulate the tumor growth compared to vehicle; radiation alone resulted in a significant decrease in tumor growth rate. LLY-283 in combination with radiation significantly increased the tumor growth delay compared to vehicle and to radiation alone. This in vivo treatment protocol was extended to the pancreatic carcinoma cell line PSN1 (Fig. 5D,E). For PSN1 tumors, the absolute growth delay for LLY-283 alone was 1.5 ± 1.17 days, and radiation alone was 6.62 ± 0.78 days. Mice treated with the combination of LLY-283 and radiation showed an absolute growth delay of 13 ± 1.3, which exceeded the sum of the growth delays of the individual treatments. Based on the absolute growth delays, the DEF for PSN1 tumors was 1.85. The log-rank analysis of tumor growth to 1000mm^3^ revealed that LLY-283 alone did not significantly modulate the tumor growth compared to vehicle; radiation alone resulted in a significant decrease in tumor growth rate. The combination of LLY-283 with radiation significantly increased the PSN1 tumor growth delay compared to vehicle and to radiation alone. Altogether, these data suggest that LLY-283 enhances the in vivo radiosensitivity of human tumor cells.

## Discussion

PRMT5 dimethylated proteins participate in a number of regulatory pathways that can contribute to tumor development and progression implicating PRMT5 as a target for cancer therapy. Accordingly, inhibitors of PRMT5 are currently undergoing clinical trials as monotherapy for hematological and solid malignancies^40^. However, in addition to affecting multiple molecular processes that contribute to tumor cell survival and proliferation^41–43^, PRMT5 can also participate in the DNA damage response (DDR)^17,19,44,45^, a critical determinant of cellular radiosensitivity. As shown, knockdown of PRMT5 using siRNA enhanced the radiosensitivity of three cell lines initiated from different solid tumor types. Consistent with these findings, shRNA mediated knockdown of PRMT5 was reported to enhance the radiosensitivity of lung tumor cell lines^46^. Thus, based on this knockdown approach, PRMT5 can serve as a determinant of tumor cell radiosensitivity.

To investigate the potential of PRMT5 as a clinically relevant target for tumor radiosensitization, we used the PRMT5 inhibitor LLY-283^38^. For this study, to evaluate the efficacy of LLY-283, it was necessary to assess changes in PRMT5 activity. PRMT5 symmetrically dimethylates more than 30 proteins^2^, a number of which could be involved in radiosensitivity and, likely, in a cell dependent manner. Moreover, PRMT5 activity is regulated by not only its expression level, but by post-translational modifications, the expression of interacting proteins and the availability of factors that regulate its catalytic activity^47^. Thus, as an indicator of LLY-283 mediated inhibition we used an ELISA to assess changes in the overall levels of sDMA^22^, the product of PRMT5 activity. As shown, LLY-283 treatment of the three tumor cell lines resulted in a dose dependent decrease in sDMA levels indicative of PRMT5 inhibition. In addition, as was observed for the siRNA targeting of PRMT5, LLY-283 treatment also induced the enhancement of tumor cell radiosensitivity.

Regarding the mechanisms responsible, γH2AX and neutral comet analyses showed that LLY-283 does not influence the initial level of radiation-induced DSBs but does inhibit their repair, similar to the effects of PRMT5 knockdown on DSB induction and repair. Thus, data suggest that the enhanced radiosensitivity induced by targeting PRMT5 involves an inhibition of DSB repair. The PRMT5 inhibitor EPZ015666 was also shown to enhance the radiosensitivity of lung tumor cell lines^46^. However, in that study only one time point after irradiation was evaluated making it difficult to determine whether the radiosensitization was the result of an increase in damage or an inhibition of repair. Consistent with a role for PRMT5 in regulating gene expression^2^, LLY-283 decreased the level of mRNAs corresponding to critical components of the DDR (*Cell Cycle* and *DNA Replication, Recombination, Repair*). In addition, LLY-283 treatment modified the pattern of mRNA splicing with the alternatively spliced transcripts also enriched in genes that participate in the DDR. These results, suggest that the radiosensitization induced by LLY-283, at least in part, is mediated by a decrease in the expression and/or splicing of DDR related genes.

The clinical applicability of a radiosensitizing agent depends, at least in part, on the preferential sensitization of tumor cells over normal cells. Treatment of the normal human fibroblast cell line MRC9 with LLY-283 reduced PRMT5 activity as reflected by sDMA levels to a similar degree as in the tumor cells. However, in contrast to tumor cells, the PRMT5 inhibitor had no effect on the radiosensitivity of normal fibroblasts. This differential radiosensitization may involve the significantly lower level of PRMT5 activity shown in the normal fibroblast cells as compared to the tumor cell lines, which is in line with previous reports showing that PRMT5 activity and/or expression is typically elevated in tumor versus normal cells^43,48–50^. Moreover, PRMT5 is consider as an oncogene in the context of its ability to repress expression of some tumor suppressor genes and regulate signaling molecules at a post-translational level^51,52^. This oncogenic role may also provide a possible mechanism for the differential radiosensitization of tumor and normal cells. A weakness of this study is that only one normal cell line was evaluated.

A process critical to the preclinical evaluation of putative radiosensitizing agents is the extension of in vitro analyses to an in vivo tumor xenograft model. In the initial preclinical study of LLY-283 as a radiosensitizer described here, a single dose reduced tumor sDMA levels for at least 24 h, indicating the effective targeting of PRMT5 under in vivo conditions. Combining LLY-283 with a 3-day fractionated irradiation protocol then resulted in a significant increase in the radiation-induced tumor growth delay, indicative of in vivo radiosensitization. Thus, these data suggest that delivery of this PRMT5 inhibitor in combination with radiotherapy may improve tumor treatment response.

### Supplementary Information

Supplementary Figures.

## Data Availability

Gene expression data generated in this study are publicly available in gene expression omnibus (GEO) GSE264609 (https://www.ncbi.nlm.nih.gov/geo/query/acc.cgi?acc=GSE264609). All other data generated or analyzed during this study are included in this published article (and its Supplementary Information files).
