# Supplementary material for: Targeting PRMT5 enhances the radiosensitivity of tumor cells grown in vitro and in vivo
Source: Sci Rep. 2024 Jul 27;14:17316. doi: 10.1038/s41598-024-68405-8 (PMC11283541; doi:10.1038/s41598-024-68405-8)
Supplement: Supplementary file 1 — Supplementary Figures. [file 41598_2024_68405_MOESM1_ESM.pdf]

Targeting PRMT5 enhances the radiosensitivity of tumor cells grown *in vitro* and *in vivo*

Charlotte Degorre[1], Steven Lohard[1], Christina N. Bobrek[1], Komal N. Rawal[1], Skyler Kuhn[2], Philip J. Tofilon[1*].

Supplementary information S1

Uncropped western blots and corresponding ladder for Figure 1.

Figure title: Effects of PRMT5 knock-down on the radiosensitivity of tumor cell lines.

Authors: Charlotte Degorre[1], Steven Lohard[1], Christina N. Bobrek[1], Komal N. Rawal[1], Skyler Kuhn[2], Philip J. Tofilon[1].

Ladder reference: Chameleon Duo - 928-600000

U251

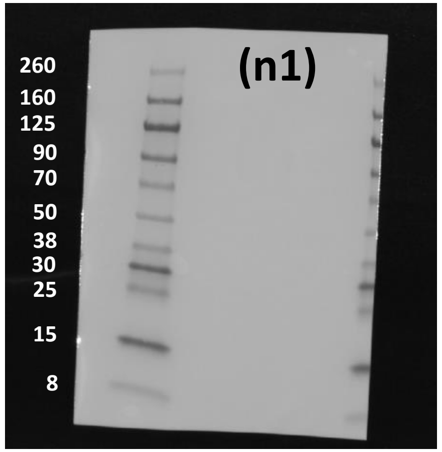

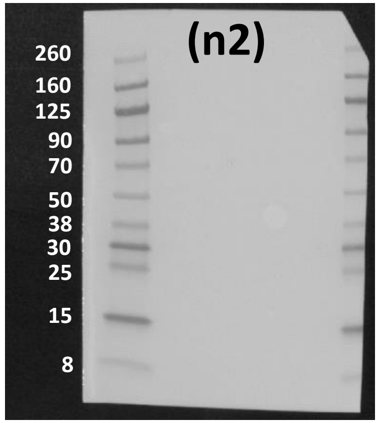

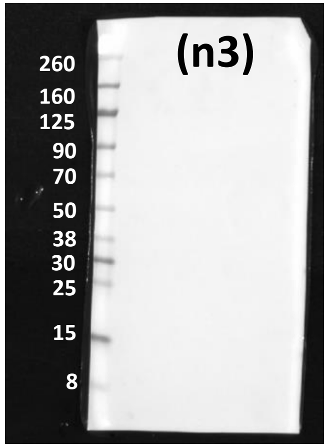

Ladder reference: Precision Plus Protein Dual Color Standards - 1610374

PSN1

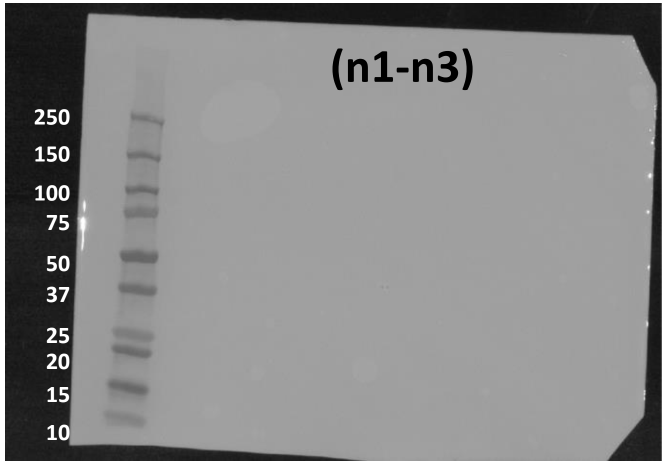

Ladder reference: Precision Plus Protein Dual Color Standards - 1610374

MDA-MB-231

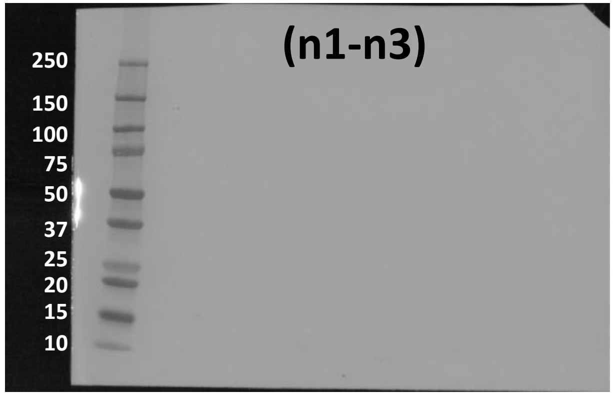

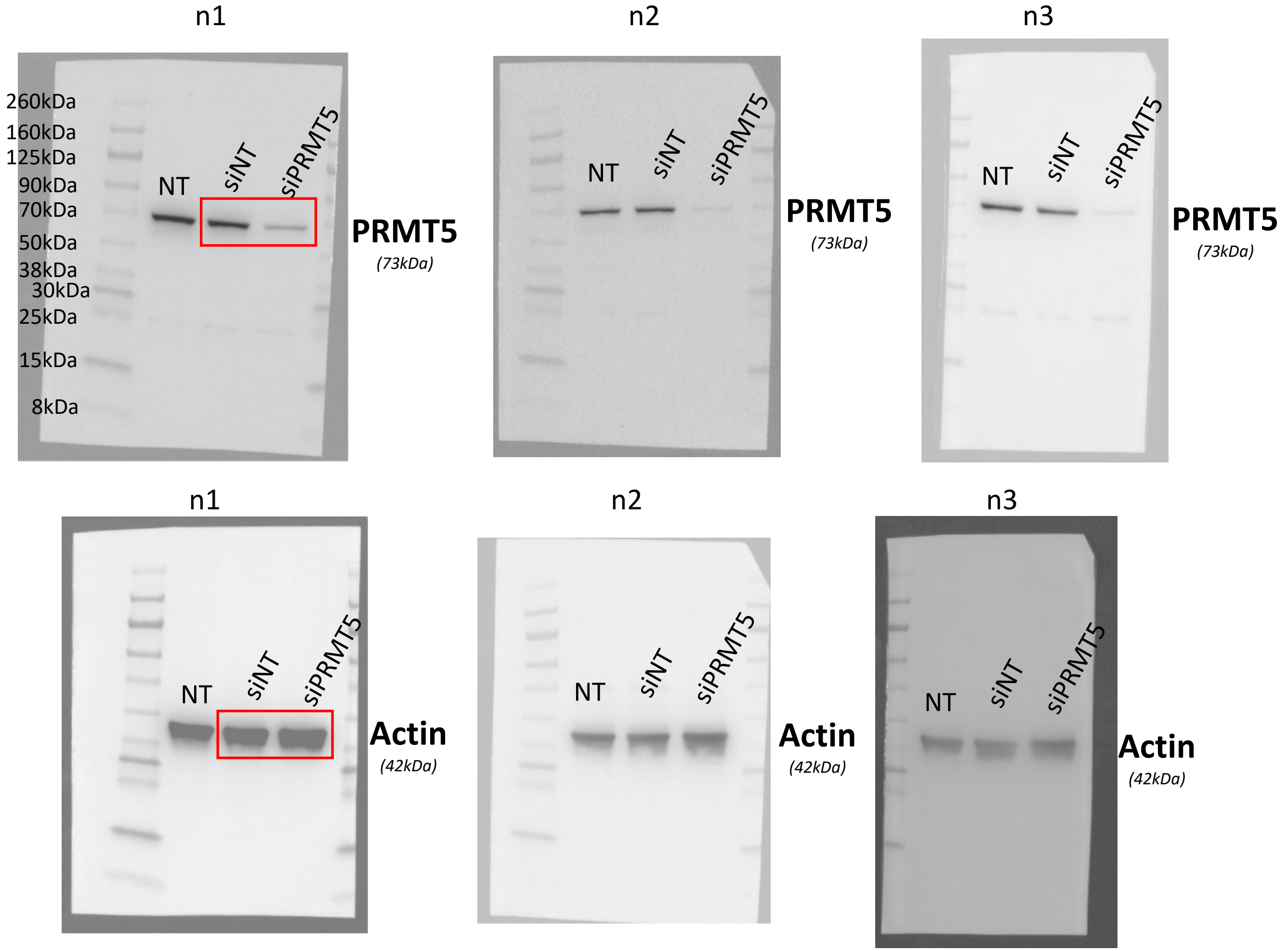

Three biological replicates of western blot experiment. Biological replicates for the U251 cell line were run on three different gels.

Primary antibodies:
- PRMT5: Cell signaling #79998
- Actin: Millipore #MAB1501

PRMT5 was detected with a Goat anti-rabbit-HRP antibody (Cell signaling #7074) and Actin with a Goat anti-mouse-HRP antibody (Cell signaling #7076). Image were acquired using the Biorad imager (ChemiDoc MP imaging system)

PSN1

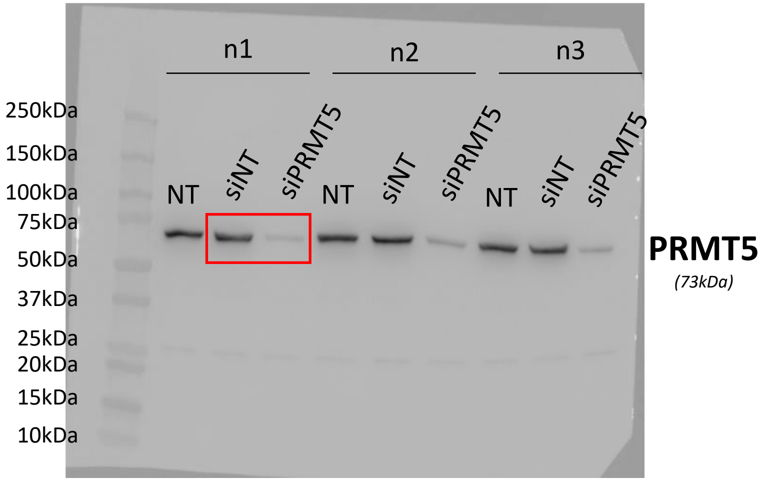

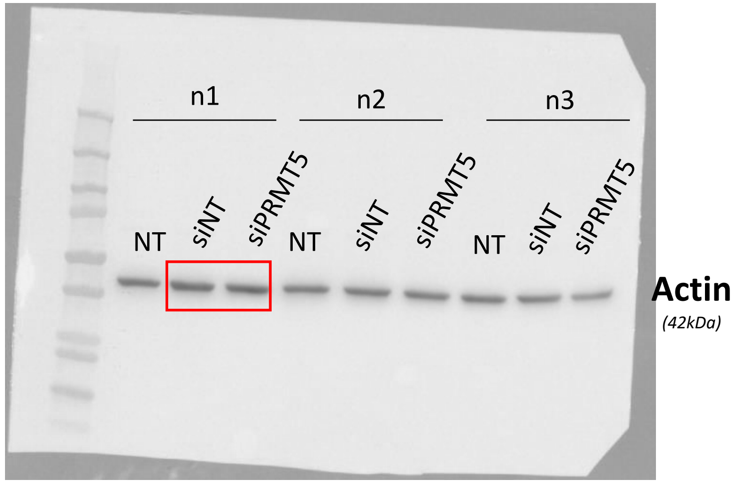

MDA-MB-231

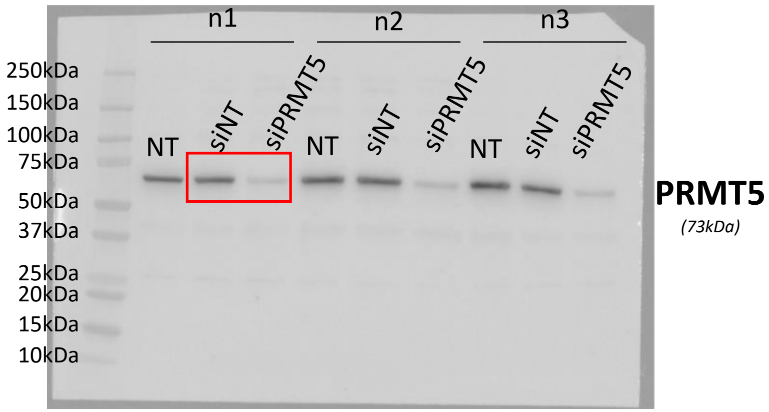

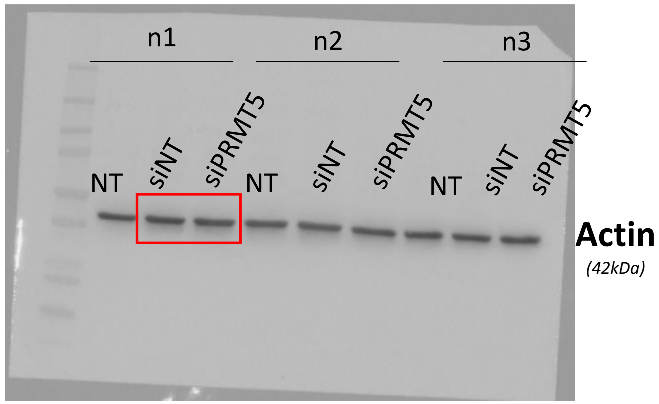

Three biological replicates of western blot experiment. All the biological replicates for PSN1 and MDA-MB-231 were run on the same gel.

Primary antibodies:
- PRMT5: Cell signaling #79998
- Actin: Millipore #MAB1501

PRMT5 was detected with a Goat anti-rabbit-HRP antibody (Cell signaling #7074) and Actin with a Goat anti-mouse-HRP antibody (Cell signaling #7076). Image were acquired using the Biorad imager (ChemiDoc MP imaging system)

Supplementary information S2

siPRMT5 decrease sDMA level in tumor cell lines

Authors: Charlotte Degorre[1], Steven Lohard[1], Christina N. Bobrek[1], Komal N. Rawal[1], Skyler Kuhn[2], Philip J. Tofilon[1].

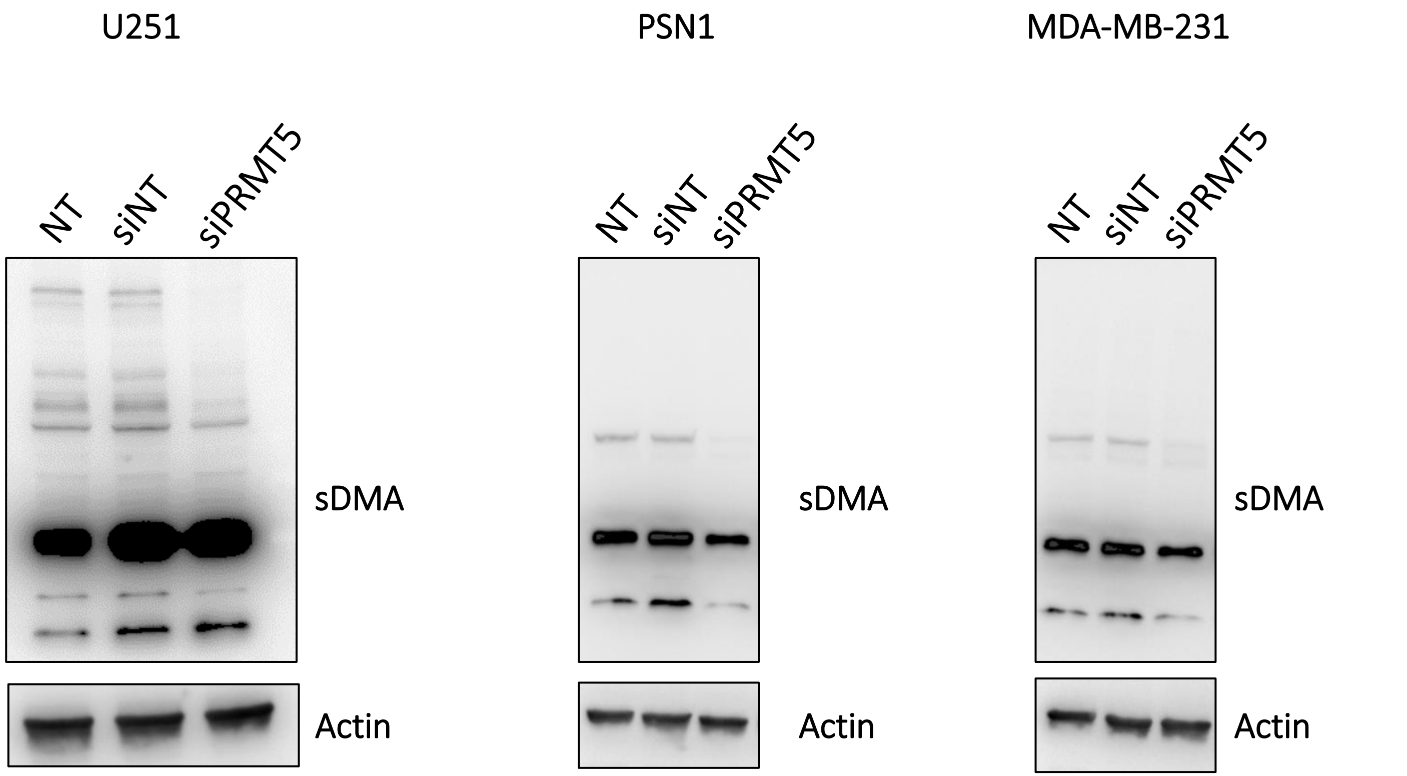

Supplementary figure S2: siPRMT5 decrease sDMA level in tumor cell lines. Tumor cell lines were transfected with non-targeting siRNA (siNT) or siRNA against PRMT5 (siPRMT5). 48 hours post-transfection, cell lysates were immunoblotted for PRMT5 and Actin as a loading control.

Supplementary information S3

Growth curves for individual mice in each treatment group.

Authors: Charlotte Degorre[1], Steven Lohard[1], Christina N. Bobrek[1], Komal N. Rawal[1], Skyler Kuhn[2], Philip J. Tofilon[1].

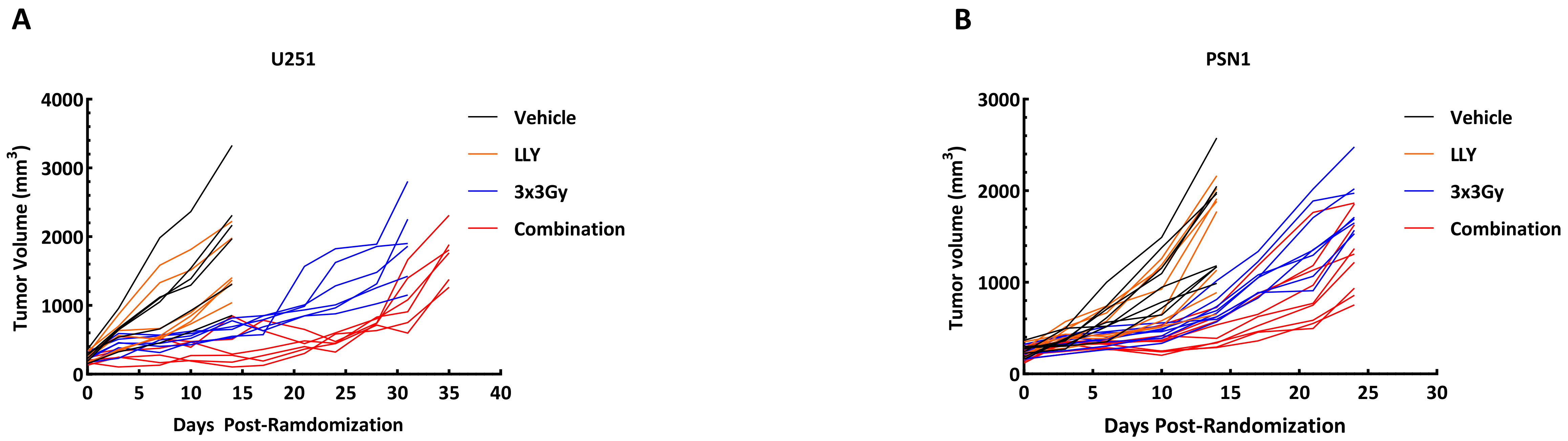

Supplementary Figure S3: Mice bearing U251 (A) or PSN1 (B) leg xenografts were treated with 3Gy for three consecutive days and received either vehicle or LLY-283 (100 mg/kg) 24 h before irradiation by oral gavage. Tumors were measured twice a week. Graph shows the growth curves for individual mice in each treatment group. Individual curves were used to calculate the absolute growth delay.
